# A Clinical Audit on Pre-Treatment Assessment Protocol Adherence to Clozapine Therapy

**DOI:** 10.1192/bjo.2025.10619

**Published:** 2025-06-20

**Authors:** Bilawal Khalid, Qumar Shahzad, Ali Anjum

**Affiliations:** 1 Punjab Institute of Mental Health, Lahore, Pakistan; 2 Noor Hospital, Lahore, Pakistan

## Abstract

**Aims:** This study meticulously evaluated adherence to the pre-clozapine initiation protocols outlined in The Maudsley Prescribing Guidelines in Psychiatry, 13th Edition. The study, conducted at different psychiatry units of the Punjab Institute of Mental Health Lahore, Pakistan, aims to evaluate the comprehensiveness of baseline evaluations and uncover significant deficiencies in monitoring vital physical health markers (like ECG) crucial for patient safety and treatment effectiveness.

1. In this study the clinical practices of pre-clozapine are critically assessed based on standard guidelines

2. It delineates the systemic, clinical, and administrative impediments affecting adherence to pre-clozapine workup protocols.

3. The completeness, accuracy, and consistency of documentation are ensured during the study.

**Methods:** This retrospective analysis examined case notes from 42 patients to evaluate compliance with pre-clozapine workup protocols at the Punjab Institute of Mental Health, Lahore. The data was examined in accordance with The Maudsley Prescribing Guidelines in Psychiatry, 13th Edition, to evaluate clinical practices, identify hurdles to adherence, and assess trends in the completion of assessments and the accuracy of documentation.

**Results:** The baseline assessments included a full blood count, liver function test, urea and electrolytes analysis, and plasma glucose measurement, all of which were conducted in 100% of cases. However, electrocardiography (ECG) was performed in only 76% of patients before clozapine initiation. Blood lipid profiling was completed in 33% of cases, while erythrocyte sedimentation rate (ESR) and plasma troponin assessments were conducted in only 19% and 14% of cases, respectively. Notably, C-reactive protein (CRP), beta-natriuretic peptide, and general physical examinations were entirely absent from the records, highlighting significant gaps in baseline cardiovascular and haematological risk assessments.

**Conclusion:** This audit identified significant gaps in pre-clozapine workups at the Punjab Institute of Mental Health, Lahore, including protocol deficiencies, inadequate staff/doctors training, sampling errors, and inconsistencies in prescriber practices. Communication breakdowns among participants and administrative constraints, such as funding and staffing limitations. To address these challenges, the implementation of standardized protocols, enhanced staff/doctor training, improved participants’ communication/documentation, adequate resource allocation, and quality assurance measures. Strengthening these areas is critical to ensuring a comprehensive and safe clozapine initiation therapy.

